# Tailoring of polar and nonpolar ZnO planes on MgO (001) substrates through molecular beam epitaxy

**DOI:** 10.1186/1556-276X-7-184

**Published:** 2012-03-09

**Authors:** Hua Zhou, Hui-Qiong Wang, Xia-Xia Liao, Yufeng Zhang, Jin-Cheng Zheng, Jia-Ou Wang, Emin Muhemmed, Hai-Jie Qian, Kurash Ibrahim, Xiaohang Chen, Huahan Zhan, Junyong Kang

**Affiliations:** 1 Key Laboratory of Semiconductors and Applications of Fujian Province, Department of Physics, Xiamen University, Xiamen, 361005, People's Republic of China; 2 State Key Lab of Silicon Materials, Zhejiang University, Hangzhou, 310027, People's Republic of China; 3 Beijing Synchrotron Radiation Facility, Institute of High Energy Physics, Chinese Academy of Sciences, Beijing, 100049, People's Republic of China

**Keywords:** ZnO, MgO, polar, nonpolar, RHEED, XRD, XAS

## Abstract

Polar and nonpolar ZnO thin films were deposited on MgO (001) substrates under different deposition parameters using oxygen plasma-assisted molecular beam epitaxy (MBE). The orientations of ZnO thin films were investigated by *in situ *reflection high-energy electron diffraction and *ex situ *X-ray diffraction (XRD). The film roughness measured by atomic force microscopy evolved as a function of substrate temperature and was correlated with the grain sizes determined by XRD. Synchrotron-based X-ray absorption spectroscopy (XAS) was performed to study the conduction band structures of the ZnO films. The fine structures of the XAS spectra, which were consistent with the results of density functional theory calculation, indicated that the polar and nonpolar ZnO films had different electronic structures. Our work suggests that it is possible to vary ZnO film structures from polar to nonpolar using the MBE growth technique and hence tailoring the electronic structures of the ZnO films.

**PACS: **81; 81.05.Dz; 81.15.Hi.

## Background

ZnO film has attracted much attention due to its various applications such as short wavelength lasers, vacuum fluorescent or field-emission displays, high-power high-frequency devices, and light-emitting diodes [1-4]. High-quality ZnO films are usually grown on expensive and hexagonal substrates such as ZnO, GaN, and sapphire and tend to be polarized, leading to built-in electric field in device structures known as the quantum-confined Stark effect [5]. To overcome this disadvantage, there is an emerging interest of growing nonpolar ZnO thin films. When ZnO thin films are deposited on the cubic substrate of MgO (100), nonpolar m-plane (10-10) and polar c-plane (0001) of ZnO can be grown by molecular beam epitaxy (MBE) and pulsed laser deposition, respectively [6]. In this work, we show that both polar and nonpolar ZnO thin films can be grown on MgO (001) substrates using oxygen plasma-assisted MBE. It is found that the electronic properties of ZnO films are different between polar and nonpolar structures.

## Methods

The MgO substrates were first degreased by ultrasonic bath in acetone, followed by ethanol. After being introduced into the MBE growth chamber (ultra-high vacuum environment, with a base pressure of 10^-9 ^mbar), the substrates were annealed at 420°C for 60 min while the power of the radiofrequency plasma source was set to 250 W and the oxygen partial pressure maintained at 5 × 10^-5 ^mbar. The detailed experimental process of plasma-assisted MBE can be found elsewhere [6,7]. During growth of ZnO films, the temperature of elemental zinc source (with a purity of 99.99999%) was maintained at 330°C, and the oxygen partial pressure was kept at 1 × 10^-5 ^mbar with the power of plasma source at 180 W. All the films reported in this work were grown at these conditions for 60 min. The only growth parameter that changed for each film is the substrate temperature, which ranges from 100°C to 480°C. *In situ *reflection high-energy electron diffraction (RHEED) was used to examine the surface structure of the MgO substrate (before depositing ZnO) and the ZnO films (after the deposition). The film roughness was characterized by atomic force microscopy (AFM). The polar and nonpolar structures of the ZnO films were determined by X-ray diffraction (XRD) using a Cu anode (*λ*_Kα1 _= 1.54056 Å). The electronic structures of the thin films were probed by synchrotron-based X-ray absorption spectra (XAS). The component analysis of XAS was done through first principles' all-electron calculations based on density functional theory (DFT) [8] using generalized gradient approximations [9], as implemented in the Wien2k package (Vienna, Austria) [10]. A dense k-point mesh of 22 × 22 × 12 was used to obtain a well-converged charge density, and the projected density of states (PDOS) of ZnO was calculated and compared with the experimental XAS spectra.

## Results and discussion

**Figure 1:**
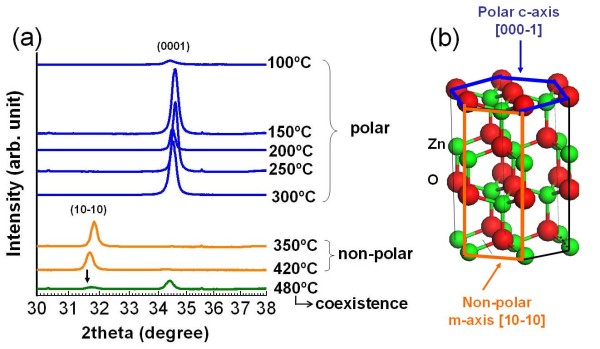
**XRD results**. (**a**) XRD plots for ZnO films grown at substrate temperature from 100°C to 480°C. (**b**) The wurtzite ZnO structure model with the illustrations of (0001) and (10-10) planes, corresponding to the structure shown in (a).

Figure 1a shows the XRD spectra of the ZnO films grown on the MgO (001) substrates with the substrate temperature varying from 100°C to 480°C. The spectra clearly show two main features: the m-plane (10-10) of ZnO with peak positions at around 31.7° and the c-plane (0002) of ZnO with peak positions at around 34.5°. When the substrate temperature is below 300°C, only the peak representing the (0002) plane orientation is visible; in the temperature range of 350°C to 420°C, the orientation of the ZnO film switches to (10-10). Interestingly, when the temperature is increased to 480°C, both the c- and m-planes exist. According to the orientations of the polar (0001) and nonpolar (10-10) planes as illustrated in Figure 1b, these results indicate that the polarization of the ZnO film can be controlled through the substrate temperature.

**Figure 2:**
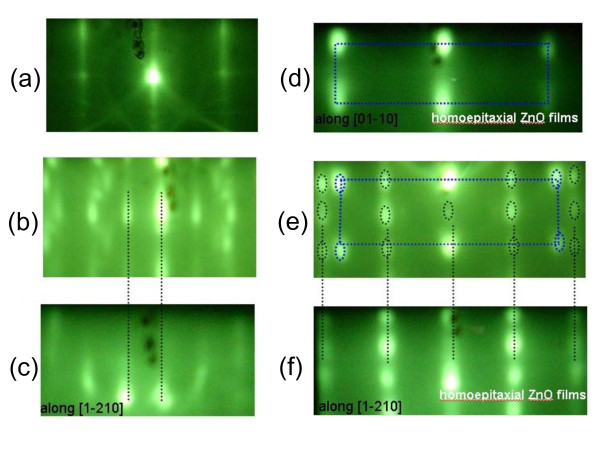
**RHEED results**. (**a**) RHEED pattern of the MgO (001) substrate prior to the ZnO film growth along the [001] azimuth. (**b**) RHEED pattern of the ZnO film surface grown at 420°C along the [1-210] azimuth. (**c**) RHEED pattern of the nonpolar single crystal ZnO surface along the [1-210] azimuth. (**d**) RHEED pattern of the polar homoepitaxial ZnO film surface along the [1-10] azimuth. (**e**) RHEED pattern of the ZnO film surface grown at 100°C, which shows the combination of the [1-10] and [1-210] azimuths. This indicates of the coexistence of two domains. (**f**) RHEED pattern of the polar homoepitaxial ZnO film polar surface along the [1-210] azimuth.

The orientation preference of the ZnO film grown at different substrate temperatures can also be verified through the RHEED patterns captured *in situ *prior to and after the growth, as shown in Figure 2. Prior to the growth, RHEED of the annealed MgO substrate shows a streaky (1 × 1) pattern along the [001] azimuth (Figure 2a). Figure 2b, e shows the RHEED patterns of the ZnO films grown at substrate temperatures of 420°C and 150°C, respectively. Both patterns are compared to those of a ZnO single crystal with nonpolar plane (Figure 2c) and a homoepitaxial ZnO film with the polar (0001) plane (Figure 2d, f). Along the [1-210] azimuth, the main feature in the RHEED pattern in Figure 2b shows an inter-streak distance close to that of the RHEED pattern from the ZnO single crystal (10-10) plane (the dotted black lines are drawn for guidance). The RHEED pattern in Figure 2e is likely to be diffracted from two sets of lattice units. One set agrees with the RHEED pattern along the [10] azimuth from the homoepitaxial ZnO (0001) film shown in Figure 2d (guided by the dotted blue lines). The other set is consistent with the RHEED pattern along the [1-210] azimuth from the same homoepitaxial ZnO (0001) film shown in Figure 2f (guided by the dotted black lines). This indicates that the polar thin film is composed of two grains that are rotated 30° from each other. Both of these grains have the same z-direction along [0001] and thus show the same XRD peak position in Figure 1a.

**Figure 3:**
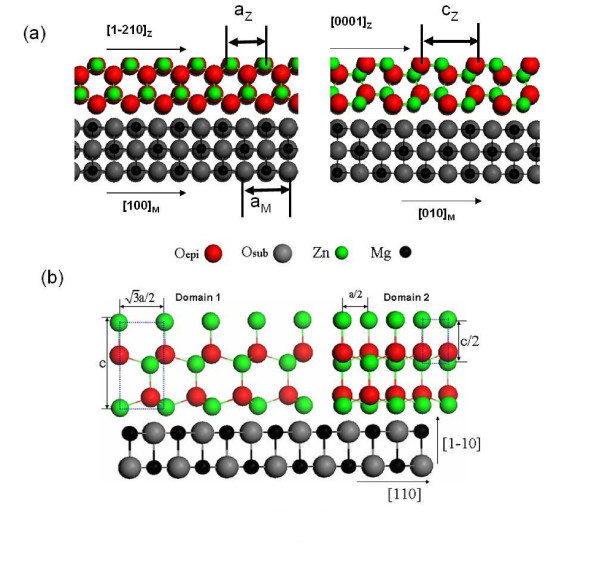
**Structural models**. (**a**) The schematic model from the MgO substrate to the nonpolar ZnO surface layer. (**b**) The schematic model from the MgO substrate to the polar ZnO surface layer.

Figure 3 illustrates the models of the plane registry between the MgO substrate and the nonpolar ZnO surface layer (Figure 3a) and between the MgO substrate and the polar ZnO surface layer (Figure 3b). The gaps in between the substrates and the surface layers imply that there could be some interfacial structural configurations that are not detectable by RHEED and XRD. However, it is worth pointing out that the spotty RHEED patterns in Figure 2 feature the three-dimensional thin film growth mode. In fact, the ratio of the horizontal and vertical lattice sizes in Figure 3 agrees well with the ratio of the vertical and horizontal inter-spot distances in the RHEED patterns shown in Figure 2.

**Figure 4:**
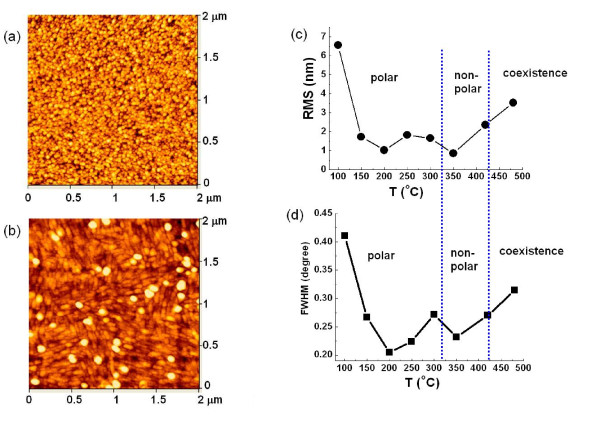
**AFM results**. (**a**) AFM image of the ZnO film surface grown at 150°C. (**b**) AFM image of the ZnO film surface grown at 420°C. (**c**) The evolution of surface roughness as a function of substrate temperature. (**d**) The evolution of XRD FWHM values as a function of substrate temperature.

The surface morphologies of the polar and nonpolar films are also studied by AFM. In Figure 4a, the AFM image of the ZnO film grown at 150°C shows the even distribution of nanoparticles. On the other hand, the AFM image of the film grown at 420°C is more 'stripe'-like. These features are an obvious difference and are in agreement with previous reports [11,12], which further confirms the structural observations from XRD and RHEED. Figure 4c, d plots the evolution of thin film roughness determined by AFM and the corresponding full width at half maximum (FWHM) values from XRD features as the growth temperature increases from 100°C to 480°C. The polar and nonpolar temperature regions are separated by the vertical dash lines. The trends shown in these two plots are in excellent agreement, in light of the fact that the smaller FWHM values indicate the larger grain size and hence the smaller roughness. It is shown that the most favorable substrate temperatures are 200°C and 350°C for polar and nonpolar growths (in order to obtain the smallest surface roughness), with the FWHM values of 0.21° and 0.23°, respectively. Although these FWHM values are considered fairly large, which indicates small grain sizes in the films, it is still possible to maintain bulk-like optoelectronic properties for the films [13].

**Figure 5:**
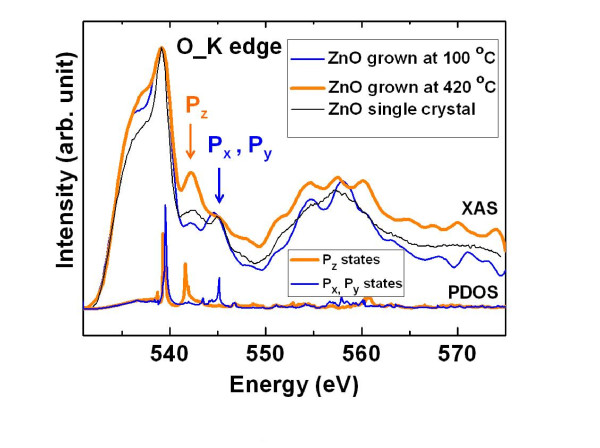
**XAS results**. XAS O-K edge spectra for the polar and nonpolar ZnO compared with PDOS from DFT calculations.

The electronic structures of the grown polar and nonpolar ZnO films are also studied by synchrotron-based XAS. The oxygen K-edge spectra of the ZnO films are shown in Figure 5. The blue curve is from the polar film grown at 100°C, and the thicker orange curve is from the nonpolar film grown at 420°C. For comparison, the XAS spectrum of the polar ZnO single crystal is also presented as the thinnest black curve. As discussed in other studies [14,15], in the photon energy region of approximately 530 to 539 eV, the XAS feature can be mainly assigned to the states of O 2*p *hybridized with Zn 4*s*. In the region of approximately 539 to 550 eV, the spectrum is mainly attributed to O 2*p *hybridized with Zn 4*p *states. Above 550 eV, the contribution mainly comes from the O 2*p *and Zn 4*d *mixed states. As illustrated in Figure 5, the spectrum feature of the polar ZnO film is close to that of the polar ZnO single crystal. The major differences between the polar and nonpolar ZnO films appear in the region of approximately 539 to 550 eV, which is originated from the hybridization of O 2*p *and Zn 4*p *states. Such difference has also been reported previously by Guo et al. [16] for an XAS study of highly oriented ZnO microrod arrays with two different beam incidence angles: one along the [000-1] axis and the other along its orthogonal axis. This is because of the lack of center of inversion in the wurtzite structure, which induces an inherent asymmetry. As shown in the PDOS (lower layer of Figure 5) from DFT calculations, the peak positions contributed by oxygen *p_z _*or *p_x _*(*p_y_*) orbital are well separated at about 12 and 15 eV above Fermi energy, which correspond to the binding energies of 542 and 545 eV, respectively. Thus, these peaks can serve as signatures for distinguishing different oxygen *p *orbital components. When the XAS spectrum for the polar thin film at 100°C (blue curve) is being collected, the beam impinges on the sample along the *c*-axis direction as shown in Figure 1b; the peak at 545 eV is stronger (upper layer of Figure 5), featuring the excitation to the *p*_*x*_, and *p_y _*states (as compared with the DFT calculations). For the nonpolar film sample, the beam incidence is along the *m*-axis, and the excitation to the *p_z _*states is enhanced. The deposited films are grown in Volmer-Weber mode (island style) similar to the highly oriented and anisotropic ZnO microrod [16]. The agreement between our results and the report in another study [16] further confirms our assessment that the polar and nonpolar structure of ZnO films are grown at 100°C and 420°C, respectively.

## Conclusions

In summary, ZnO thin films were grown at varying substrate temperatures while keeping other growth parameters unchanged. Structural characterization by XRD revealed a sharp (10-10) peak at 31.7° in the 2*θ *scanning for films grown between 320°C and 420°C, indicating the formation of nonpolar m-plane. For films grown below 320°C, a strong (0002) peak at 34.5° representing polar c-plane was instead identified; for films grown above 420°C, nonpolar and polar planes coexist. *In situ *RHEED measurements also confirmed that the grown ZnO thin films were mostly of the wurtzite phase. In addition, the polar ZnO film structure was composed of two domains with a rotation angle of 30°. AFM images showed that the roughness of the thin film surface changed with the substrate temperature and was coupled with the evolution of the FWHM values from XRD. The fine structures of XAS spectra indicated that the polar and nonpolar ZnO films had different electronic structures, which was consistent with the microstructure observations and DFT calculations. Our work suggests the possibility of motoring polar and nonpolar ZnO films using the MBE growth technique and hence tailoring the electronic structure of the ZnO films.

## Abbreviations

AFM: atomic force microscopy; DFT: density functional theory; FWHM: full width at half maximum; MBE: molecular beam epitaxy; PDOS: projected density of state; RHEED: reflection high energy electron diffraction; XAS: X-ray absorption spectroscopy; XRD: X-ray diffraction.

## Competing interests

The authors declare that they have no competing interests.

## Authors' contributions

HZ carried out the growth experiments and drafted the manuscript. H-QW led the project, analyzed the results, and revised the manuscript. X-XL, YFZ, J-CZ, J-OW, EM, H-JQ, and KI performed the synchrotron-based XAS experiments and analyze the data. J-CZ also conducted the DFT calculations. XHC, HHZ, and JYK participated in the MBE experiments and discussions. All authors read and approved the final manuscript.
